# Machine learning (ML)-assisted surface tension and oscillation-induced elastic modulus studies of oxide-coated liquid metal (LM) alloys

**DOI:** 10.1088/2515-7639/acf78c

**Published:** 2023-09-26

**Authors:** Kazi Zihan Hossain, Sharif Amit Kamran, Alireza Tavakkoli, M Rashed Khan

**Affiliations:** 1 Department of Chemical & Materials Engineering, University of Nevada, Reno, NV, United States of America; 2 Department of Computer Science & Engineering, University of Nevada, Reno, NV, United States of America

**Keywords:** gallium alloys, machine learning, surface tension, Tensiometry, pendant droplets, elastometry, liquid metal

## Abstract

Pendant drops of oxide-coated high-surface tension fluids frequently produce perturbed shapes that impede interfacial studies. Eutectic gallium indium or Galinstan are high-surface tension fluids coated with a ∼5 nm gallium oxide (Ga_2_O_3_) film and falls under this fluid classification, also known as liquid metals (LMs). The recent emergence of LM-based applications often cannot proceed without analyzing interfacial energetics in different environments. While numerous techniques are available in the literature for interfacial studies- pendant droplet-based analyses are the simplest. However, the perturbed shape of the pendant drops due to the presence of surface oxide has been ignored frequently as a source of error. Also, exploratory investigations of surface oxide leveraging oscillatory pendant droplets have remained untapped. We address both challenges and present two contributing novelties- (a) by utilizing the machine learning (ML) technique, we predict the approximate surface tension value of perturbed pendant droplets, (ii) by leveraging the oscillation-induced bubble tensiometry method, we study the dynamic elastic modulus of the oxide-coated LM droplets. We have created our dataset from LM’s pendant drop shape parameters and trained different models for comparison. We have achieved >99% accuracy with all models and added versatility to work with other fluids. The best-performing model was leveraged further to predict the approximate values of the nonaxisymmetric LM droplets. Then, we analyzed LM’s elastic and viscous moduli in air, harnessing oscillation-induced pendant droplets, which provides complementary opportunities for interfacial studies alternative to expensive rheometers. We believe it will enable more fundamental studies of the oxide layer on LM, leveraging both symmetric and perturbed droplets. Our study broadens the materials science horizon, where researchers from ML and artificial intelligence domains can work synergistically to solve more complex problems related to surface science, interfacial studies, and other studies relevant to LM-based systems.

## 1. Introduction

Herein, we demonstrate two methods that can be harnessed to study the elastic modulus and the surface tension- two of the most important properties of gallium alloys. Numerous techniques are currently available in the literature to study the elastic modulus and surface tension of gallium alloys; however, the utility of pendant droplets (symmetric and asymmetric) for (a) machine-learning (ML) assisted surface tension and (b) oscillation-induced elastic modulus studies have remained unexplored. Gallium and its alloys at low melting points [1–3] have emerged as the most promising soft conductor for sensors, actuators, pumps, flexible electronics, and other microfluidics applications [3–10]. High electrical conductivity, low toxicity, low vapor pressure, biocompatibility, and tunable surface tension (∼600 mN m^−1^–100 mN m^−1^) are some of the notable properties at room temperature [11, 12]of these alloys; however, the unique behavior of these alloys arise from their native 1–5 nm thick, and passivating Ga_2_O_3_ layer [13]. Such a thin layer of semisolid surface oxide forms rapidly and does not grow thicker in air, even at a very low oxygen concentration (∼1 ppm) [2], providing the mechanical strength to produce different functional patterns and structures [4, 14]. In contrast, the presence of oxide reduces the surface tension of the bulk metal [15, 16], and because of that, the electrohydrodynamic methods [17] of tuning the thickness of the oxide have gained significant research interest over the last few years [18–23].

Numerous techniques are currently presented in the literature to characterize surface tension of the oxide- i.e. pendant drop or sessile drop methods [24, 25], cone and plates in rheometers [26], parallel plates techniques [12, 14, 24, 27] in conjunction with the methods available for common fluids, i.e., capillary rise [28, 29], Wilhelmy plate [30, 31], Du Noüy ring [32, 33], maximum bubble pressure [34–36], levitated drop [37, 38], and capillary wave scattering methods [11, 39, 40]. Among these many techniques, the pendant and sessile drop-based techniques are the most convenient ways to measure the surface tension of liquids at room temperature. Pendant drop-based analyses are often preferred over sessile drops because of more control over the control volume and studies involving the surrounding environment; however, surface tension measurement of gallium alloys using pendant drops is often very challenging due to the presence of the Ga_2_O_3_ layer in the air (i.e., due to surface oxidation). Pendant drops of high-surface tension fluid with an oxide coating also tend to produce perturbed and distorted shapes that impede surface tension measurements [26, 41]. Eutectic gallium indium (eGaIn) or eutectic gallium indium tin (Galinstan) are high-surface tension gallium alloys often exhibiting such challenges.

Functional studies of eGaIn or Galinstan frequently cannot proceed without interfacial and intermediate surface tension measurements in different environments. Different commercial and open-source software can be utilized to measure the surface tension from 2D images of droplets, differentiating the background and drop shape intensity contrasts. However, the primary assumption of the pendant drop-based method is that fluid forms a symmetric droplet along the central axis of the droplets [42–44]. While this axisymmetric assumption holds for most generic fluids, nonaxisymmetric or perturbed drop shapes often limit experimental studies due to coating, surfactants, or environmental influences [45–47]. For Ga_2_O_3-_coated liquid metal (LM) droplets, the challenges of perturbed droplets remain unreported in the literature for interfacial studies or studies related to the surface coatings of LM [11]. In this study, we aim to address this challenge where we have intentionally leveraged perturbed droplets of LMs to demonstrate the novelty of the current study. While automatic commercial software failed to provide any value for LM [43] once perturbed droplets are produced, ImageJ-based [44] or open-source [44] manual and time-consuming techniques can be leveraged to gauge surface tension values of these nonaxisymmetric droplets.

In contrast, the pendant drop analysis technique has also been leveraged in the literature to investigate the dilatational moduli- elastic and viscous modulus by oscillating the droplet of generic fluids, well-known as bubble tensiometry [48–50]. Due to the lack of the ability to measure continuous surface tension values of LM, this oscillatory bubble tensiometry has rarely been harnessed to dissect the behavior of LM in the literature. Approximating the value of these non-symmetric pendant drops will help us fill the existing knowledge gap in the literature by understanding LM’s dynamic surface tension transition due to environmental interaction from perturbed to axisymmetric shape and vice versa. Prior literature also emphasized the importance of dynamic investigations to understand the unique behavior of the LM oxide layer instead of static measurements [41]. Therefore, our current study has two contributing novelties- (i) utilizing the ML technique to predict the approximate surface tension value of perturbed pendant droplets, (ii) leveraging the oscillation-induced bubble tensiometry method to study the dynamic elastic modulus of the oxide-coated LM droplets.

We have presented an immediate solution with a ML approach to predict the surface tension of perturbed or nonaxisymmetric pendant droplets. We developed a regression-based ML technique and trained the model with a variable-shaped LM pendant drop dataset. We have also made our model versatile enough to provide shape parameters as output rather than giving the final surface tension value so that it can work with other fluids. Additionally, this model can provide an approximate surface tension value for the nonaxisymmetric droplets by interpolating from typical symmetric pendant droplets. Previously, researchers have utilized different ML-based approaches to determine the surface tension of various fluids. Soori *et al* used a classification-based convolutional neural network (CNN) to predict surface tension [51]. They used Alexnet [52] and similar classification techniques to directly predict the surface tension of different concentrations of the ethanol-water mixture. However, they considered discrete surface tension value, which is not an appropriate technique for fluid-like LM, which can show surface tension ranges due to environmental influences. In another work, Gaussian process regression was done to predict the surface tension of multicomponent metallic systems. Still, it did not incorporate the gallium-based system we have targeted here [53]. Least-squares support vector machine was found to perform better among three different optimization methods that were used to predict the surface tension of pure alcohol [54]. KNN algorithm [55] and support vector machine algorithm were used to predict the binary surface tension of the ionic liquid mixture liquid [56]. Support vector machine and heuristic method were used to develop a structure-property relationship model from the molecular structure to estimate the surface tension of common liquid compounds [57]. Droplet jetting condition from the single-jet printing process was classified using a CNN based on the MobileNetV2 [58]. However, most of these studies focused on specific fluid systems, and re-training is required if another fluid needs to be analyzed. The current work is the first approach to accurately predict the drop parameters with a ML approach trained from a LM dataset, rather than conventional approach of directly estimating the surface tension. We created our own training, validation, and testing datasets of LM droplets for this study. This image-based ML technique can predict the surface tension of a pendant drop in a quick and versatile way with more than 99% accuracy. We have used three different models -ResNet50-v2, MobileNet-v2, and EfficientNet-v2 to find out the best performing models. This model can be used for other fluids also as it has the capability to predict the drop shape parameters directly. We have also measured the approximate value of the perturbed pendant droplets with our trained models as to show the direct utility of our approach. We believe the continuous interfacial measurements of the LM droplets, whether axisymmetric or perturbed, will no longer remain an untapped issue if our proposed ML approach is adopted.

**Figure 1.**
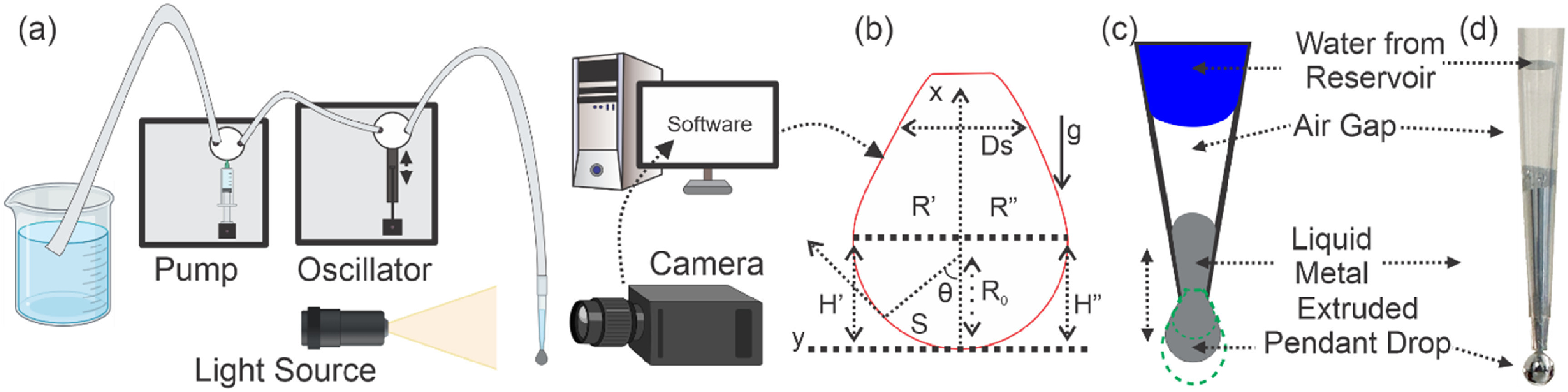
(a) Experimental setup of the goniometer to study the oscillation of a pendant drop and measure elastic and storage modulus. A pump pushes the bulk fluid and controls the extrusion from the tip. The oscillator can create oscillatory movement based on the predetermined mechanical amplitude and arm length. Fluid can be taken into the tip and extruded to create a pendant drop shape; the shape is captured with a camera using a controlled light source and analyzed with commercially available software. (b) Different geometric parameters analyzed from an axisymmetric pendant droplet. (c) Illustration of the tip extruding LM; while the oscillator moves, the water level from the reservoir moves up and down, oscillating the fluid of interest by displaying a dynamic change in the area, volume, and surface tension. (d) Side-view images of an actual tip with a pendant LM droplet.

In addition to ML-assisted surface tension study, we report a method to study elastic modulus harnessing oscillating pendant droplets- also known as ‘bubble tensiometry’ for common fluids [48, 59–61]. Pendant drop-based bubble tensiometry methods are more convenient and easily accessible to most research groups than expensive rheometers for fundamental studies. Also, this method offers a facile opportunity to manipulate the external environment of the LM droplets with precise control. Previous researchers only partially tapped the pendant drop techniques to investigate the surface tension [24]. However, we can further leverage the techniques to investigate the kinetic behavior of the LM oxide layer. We first validated our dilatational results with previous literature to compare the elastic and viscous components of the LM [12, 14, 27, 62]. As proof of concept, we also present the LM droplet turning into a softer material by showing the gradual elastic modulus reduction. Figure 1 gives a generic overview of our simplistic experimental setup, which we solely leverage for both studies.

## 2. Materials and methods

**Figure 2.**
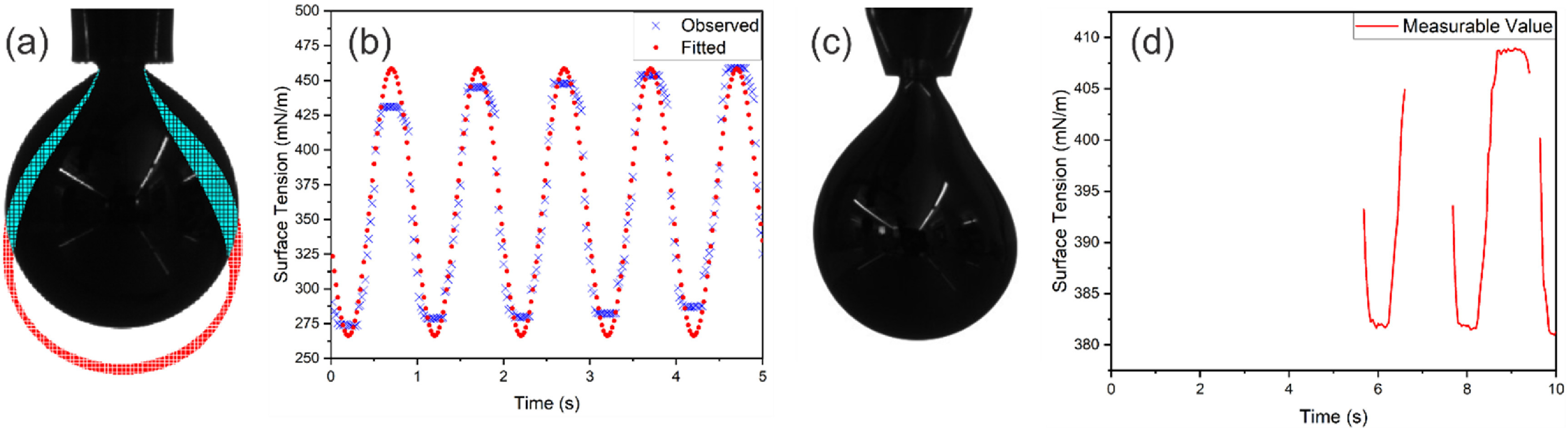
(a) Black and white image of an LM axisymmetric pendant drop directly from the DropImage software. The black color indicated the initial state of the droplets, and the blue-red lines indicated the state of the oscillatory droplets at different time points, (b) the observed surface tension data compared with predicted surface tension data based on fitted parameters, (c) a perturbed LM pendant droplet, which violates the axisymmetry assumptions; (d) measurable surface tension data over time, which is not enough to calculate the elastic and storage modulus.

Our test fluid, eGaIn, was purchased from 5N Plus Trumbull Inc. The Ramé-Hart model 500 Goniometer (Part No: 500-U4) was equipped with a camera and computer-controlled automatic dispensing system. The automatic dispenser (Part No: 100–22) worked like a syringe pump, equipped with a 250 *µ*l syringe. The software could control the flow rate by setting the Full stroke time, which was set as 30 s throughout the experimentation. The automatic oscillator (Part No: 100–28) equipped with a 50 *µ*l syringe was also bought from Ramé-Hart Instrument Co. For all the experiments, the tips used were 250 *µ*l non-sterile tips (Part No: 100-22-250T, ∼0.8 mm OD and ∼0.45 mm ID at the tip) provided by Ramé-Hart Instrument Co. and manufactured by Thermo Scientific. A representative illustration is shown in figure 1(a), and the original setup is included in the supporting section [see figure S-1]. DI water was used as reservoir fluid. Several rinsing was done to eliminate any bubbles in the tubing portion of the system. After connecting a new tip, water was not dispensed to the end of the tip. Instead, a specific portion was kept free from water to avoid any potential interaction of the LM with water, such as assisting flow behavior [63]. Fresh LM of ∼20 *µ*l was taken for each set of experiments. Around 5–10 *µ*l were extruded to conduct an oscillating experiment. The oscillator stroke length was kept constant throughout the investigation. The oscillation frequency was set as 1 Hz, and the volume-based amplitude was set as 5 *µ*l in the software. The software also provided the opportunity to set the number of recorded oscillations (Peak) during each observation which was set as 5. Therefore, we also observe five peaks in figure 2(b).

All the images were captured with the in-built U2 series camera (750 FPS SuperSpeed U2 Series Upgrade Kit, Part no: 100-12-U2, Manufactured by Ramé-Hart Instrument Co.) and analyzed with the DropImage Advanced software from Ramé-Hart [43]. The 150W Fiber Optic Illuminator (Part No: FOI-150-UL) equipped with Halogen Projector Lamp (Part No: 100-00-FOB), both sold by Ramé-Hart Instrument Co., was used as the light source to capture images. The density of air of 0.0013 g ml^−1^ and eGaIn or LM density of 6.25 g ml^−1^ were used in this study. The resolution for the pendant drop images is 480 × 640, and they are in RGB format. The dataset contains 13 081 images, of which 10 594 are for training, 1178 are for validation, and 1309 are for testing. The DropImage software could analyze these images and provide the shape parameters and surface tension value used to train the neural network. The model with the best validation result was used to final evaluate the holdout test set. Given that we are making a regression model, the ground truths for *R*_0_ and *β* were real positive numbers. The best-performing model was used to interpolate the surface tension value of the perturbed droplet.

We used oscillatory pendant drop shape analysis to measure dilatational moduli- Storage and loss modulus for LM in an air medium. Each pendant droplet’s surface tension must be measured and properly fit to the proper surface dilatational equations to determine the moduli.

### 2.1. Surface tension theory

We have adopted and modified the original theory from the literature. The pendant drop surface tension technique is based on the Young–Laplace equation shown in equation (1)—first, the density difference between the fluid of interest and the surrounding medium must be declared. Then, a fluid is generally extruded from a needle, and a pixel-calibrated camera with a constant light source is used to capture the images. Lastly, images are analyzed with intensity-based iterative image analysis techniques to fit the drop shape to the Young-Laplace equation and achieve different relevant parameters. A representative setup with parameters marked pendant droplets is shown in figure 1(b). Based on previous literature [64, 65], the detailed theory is explained in the appendix section of the supporting document, and the magnitude of these parameters is given in table S-1 and figure S-2

\begin{equation*}\gamma = \frac{{{{\Delta }}\rho gR_0^2}}{\beta }.\end{equation*}

In equation (1), *γ* is the surface tension, *Δρ* is the density difference between the study fluid and the surrounding medium, *R*_0_ is the radius shown in figure 1(b), and *g* is the gravitational acceleration constant. *β* is called the bond number or shape factor of a pendant drop and is a dimensionless number representing the ratio between the gravitational force and the surface tension force [66]. Figure 1(c) represents the setup from the perspective near the tip. An extruded pendant droplet of LM from a tip is shown in figure 1(d).

### 2.2. Oscillating droplet tensiometry

Investigating the dilatational elasticity from oscillating droplets has been utilized for different systems in the literature [48, 59–61]. The theory explained here was directly adopted from Myrvold and Hansen’s work [48] to help readers understand the concepts. We slightly modified the formula and utilized for LM system to measure the elastic and loss modulus in the air medium. The modifications in the equations will be explained in the later section. The surface elasticity, *E*, follows the definition in equation (2) provided by Gibbs,


\begin{equation*}E = \frac{{d\gamma }}{{d\ln A}}\,.\end{equation*}

Here, *A* is the surface area. The surface elasticity term indicates that *E* is a property of pure elastic surfaces. However, surfaces can have elastic and viscous components both. Therefore, ‘dilatational surface modulus’ is generally used where the contribution of this modulus depends on the relaxation process, which happens on the surface layer due to the interaction with the surrounding environments. The equilibrium (Gibbs) surface elasticity ${E_0}$ and *E* will be different for that case. The dilatational surface viscosity, ${\eta _d}$, can be defined as equation (3)


\begin{equation*}\Delta \gamma = {\eta _d}\frac{{d\ln A}}{{dt}}\,\,.\end{equation*}

Here, $\Delta \gamma $ is the surface tension difference between the expanding and equilibrium surface. ${\eta _d}$ will represent the Newtonian type of surface viscosity when elasticity is equal to zero. The complex surface dilatational modulus can be expressed as equation (4) for any other situation


\begin{equation*}{E^*} = E{^{{\prime}}} + iE{^{{\prime} ^{\prime}}}{ }.\end{equation*}

Here, $E{^{{\prime}}}$ is the storage or elastic modulus, and $E{^{{\prime} ^{\prime}}}$ is the loss or viscous modulus. In this oscillator instrument, the stroke length from a circulatory disk can be adjusted, which controls the linear up-down motion in the 50 *µ*l syringe. This linear motion changes the water level towards the tip, which varies the volume or surface area of the pendant drop with time, according to the function in equation (5)

\begin{equation*}{{\Delta {\text{ln}}}}\,A\sim \exp \left( {i\omega t} \right)\end{equation*}

where $\omega $ is the angular rate, and the loss modulus equation can be written as the following equation (6)

\begin{equation*}E^{{\prime} ^{\prime} } = \omega {\eta _d}.\end{equation*}

When, $\omega \to 0,\,E{^{{\prime}}}$ becomes ${E_0}$ and ${\eta _d}$ represent dynamic viscosity, $\eta {^{{\prime}}} = G{^{{\prime} ^{\prime}}}/\omega { }$ in a typical oscillatory rheological instrument. It is also assumed that surface area changes or area amplitude, ${A_a}$ is small. When the oscillatory droplet follows the surface area changes according to equation (5), and the consequent volume change is also small, the area changes can be represented in a sinusoidal variation presented in equation (7)

\begin{equation*}\Delta A = A - {A_0} = {A_a}\sin \left( {\omega t} \right).\end{equation*}

Here, ${A_0}$ is the equilibrium surface area. The corresponding surface tension changes can also be represented as equation (8)

\begin{equation*}\Delta \gamma = \gamma - {\gamma _0} = {\gamma _a}\sin \left( {\omega t + \delta } \right) = {\gamma _a}\sin \left( {\omega t} \right)\cos \delta + {\gamma _a}\cos \left( {\omega t} \right)\sin \delta .\end{equation*}

Here, ${\gamma _a}$ is the measured surface tension amplitude, ${\gamma _0}$ is the equilibrium surface tension, and $\delta \,$ is the phase angle. Therefore, equation (4) can be represented as equation (9)


\begin{equation*}{E^*} = E{^{{\prime}}} + iE{^{{\prime} ^{\prime}}} = \left| E \right|{\text{cos}}\delta + i\left| E \right|{\text{sin}}\delta \end{equation*}

\begin{equation*}{\text{where}},\,\left| E \right| = \frac{{{\gamma _a}}}{{{A_a}/{A_0}}}\,\,.\end{equation*}

The initially extruded pendant droplets of LM are shown in figure 2(a) with the black droplet shape. Due to the oscillatory movement of the instrument, the droplet changes its volume and area over time which are shown in blue (if the oscillated shape overlapped the area of the initial image) and red (if the oscillated shape was outside of the initially captured shape) in figure 2(a). The software, DropImage, calculated axisymmetric pendant droplets and provided the surface tension value with the associated shape parameters. The area and surface tension values over time are supposed to be fitted in equations (7) and (8) to get the ${A_0}$, ${A_a}$, ${\gamma _0}$, ${\gamma _a}$, and $\,\delta $ values to calculate the *E*′ and *E*″ values. We developed our own Python script to process the data and used the default curve_fit optimization package from the Scipy library. One such fitted curve with observed surface tension is compared in figure 2(b).

### 2.3. Modifications for LM

As LM may create perturbed or nonaxisymmetric droplets for various reasons, we must carefully modify our approach to use the pendant drop technique for LM.

#### 2.3.1. Complication with surface tension measurement

The primary assumption for pendant drop shape analysis is that a central axis will remain in the middle of the droplet, and the shape on both sides will be similar. The tip is generally excluded from the calculation by manually setting the line to restrict the region of interest. During the image analysis, the software detects the outer shape based on the intensity difference and determines the middle axis. However, this technique often fails for LM investigation by declaring sides are too different without producing any data. The distorted pendant droplet shown in figure 2(c) could not be measurable by the DropImage software. This error resulted in the discontinuous measurement shown in figure 2(d), from which proper dilatational modulus measurement is impossible. To solve this issue, we trained a neural network from the axisymmetric droplets and used them to interpolate the approximate surface tension of these perturbed or distorted droplets. These approximate values will help to measure the dilatational modulus more accurately.

#### 2.3.2. Assumptions for dilatational modulus calculation

The high perturbation during the image analysis process also resulted in unusual values, which generally created errors in the software and did not allow the software to calculate the elastic and loss modulus. Therefore, DropImage [43] was found inconsistent in producing the modulus values in the case of LM. We used our Python scripts to keep the result consistent and compared the storage and loss modulus. We excluded the perturbed data initially and fitted the equations to the only measurable values from the software.

Although we set the number of oscillations five times for each curve or observation, the instruments oscillated more and reported data of at least five continuous peaks from the whole oscillation cycle. We can see a lag from the typical sinusoidal wave if we observe the surface tension curve in figure 2(b). If we used equations (7) and (8) without modifications, the fitted amplitude values ${A_a}$ and ${\gamma _a}$ can even be negative depending on the initial phase lag. A negative amplitude value does not seem accurate as the volume increases from the initial extruded drop. Therefore, we modified the equations to include the initial lag due to the instrumental limitation, ${\delta _{ins\_lag}}$ and we got rid of that during calculations. Our modified equations used in this work are shown in equations (11) and (12)

\begin{equation*}A = {A_0} + {A_a}\sin \left( {\omega t + {\delta _{ins\_lag}}} \right)\end{equation*}

\begin{equation*}\gamma = {\gamma _0} + {\gamma _a}\sin \left( {\omega t + \delta + {\delta _{ins\_lag}}} \right).\end{equation*}

Additionally, we put constraints during the fitting procedures that ${A_0}$, ${A_a}$, ${{{\gamma }}_0}$ and ${\gamma _a}$ are always positive. The values of ${\delta _{ins\_lag}}$ and $\delta $ were set to be bound between 0 to 2 $\pi $ radians. The modulus calculations equations were used without any further modifications.

### 2.4. Deep-learning architecture

We also introduce a deep convolutional neural network (CNN) for regressing the surface tension from LM pendant droplet images. First, we elaborate on the pre-trained encoder in section 2.4.1 and the building blocks in section 2.4.2. After that, we describe the decoder module in section 2.4.3. Finally, in section 2.4.4, we discuss the objective function of the proposed architecture.

#### 2.4.1. Pre-trained encoder

**Figure 3.**
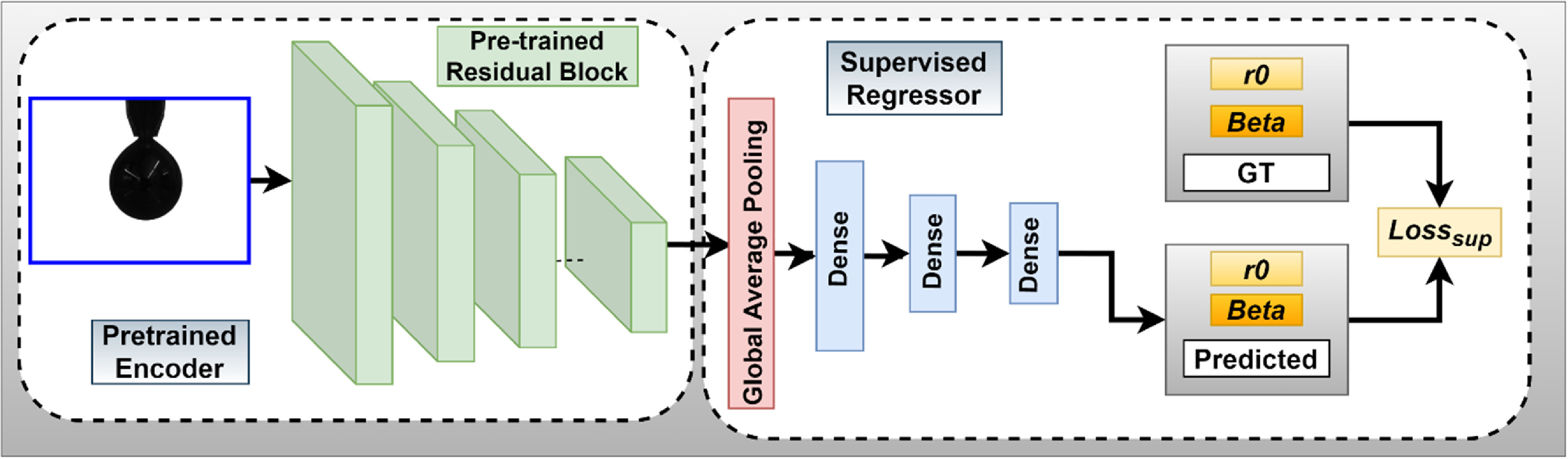
Proposed Deep Learning-based Surface Tension Regression Model. The model inputs pendant droplet images of LM and outputs both, ${R_0}$ and $\beta $ values for surface tension calculation. The model consists of a pre-trained encoder module and a supervised regressor module. We utilize mean-absolute-error for calculating the loss between predicted and ground-truth values.

**Figure 4.**
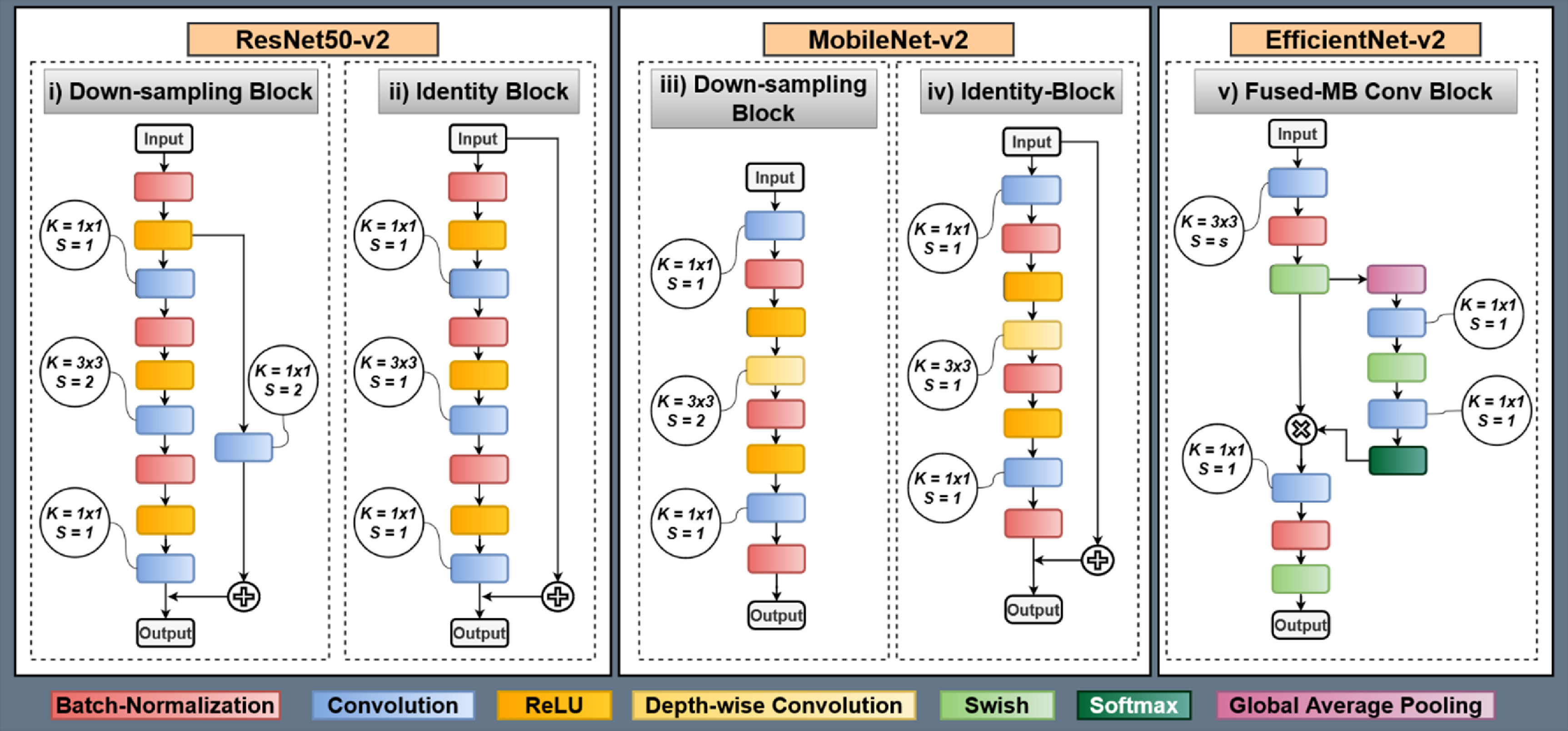
Building blocks of ResNet50-v2, MobileNet-v2, and EfficientNet-v2.

Utilizing pre-trained models for transfer learning tasks has shown tremendous promise in healthcare [67–69], physics-informed simulation [70, 71], drug discovery [72], and computational biology [73, 74]. Pre-trained models are architectures previously trained on an extensive data set, and then the weights of these models are transferred and trained on a downstream task. For example, a popular deep convolutional neural network called ResNet [75, 76] has been trained on 3.2 million ImageNet [52] images and have been used for many downstream tasks such as, predicting fluid flow [77], hydraulic fracture [78], and fluid-structure interaction [79]. Similar architectures with residual blocks have seen many adoptions in challenging downstream tasks such as image-to-image translation, image inpainting, and semantic segmentation [80, 81]. In figure 3, our proposed Deep Surface Tension Regression architecture consists of an encoder that takes the pendant droplet image as input, a decoder, and two outputs for calculating the *R*_0_ and *β* for the final surface tension calculation. The encoder consists of a pre-trained network with multiple residual and down sampling blocks, as shown in figure 4.

The basic structure of ResNet consists of a residual unit with two consecutive convolution layers and a skip connection that adds the feature tensor of the input with the output [75]. However, the authors improved upon this, utilized pre-activation with batch normalization to address the vanishing gradient problem, and proposed a new architecture called ResNet-v2 [76] with new residual and down sampling blocks. Regarding efficiency, two other architectures have proposed modified learnable blocks that utilize fewer parameters, MobileNet [82] and EfficientNet [83]. Similar to ResNet, the authors of both these models improved their architecture and proposed two new architectures, MobileNetV2 [84] and EfficientNetV2 [85]. For our experimentation, we use pre-trained encoders of these three architectures. Moreover, all of them were trained on ImageNet2012 datasets [52].

#### 2.4.2. Building blocks

First, we use the ResNet50-v2 architecture for our pre-trained encoder. The encoder consists of residual down sampling and residual identity blocks with pre-activation. We illustrate these residual blocks in figures 4(i) and (ii). The residual down-sampling blocks have three sub-blocks successively with batch-normalization, ReLU, and convolution. Also, a skip connection with the convolution layer is added from the first sub-blocks ReLU with the last convolution layer’s output. The first and last convolution has kernel size, *k* = 3, and stride, *s* = 1. The second and skip-connection convolutions have stride, *s* = 2.

Next, we use the MobileNetV2 pre-trained encoder, consisting of down-sampling and identity blocks. These blocks are visualized in figures 4(iii) and (iv). Unlike ResNetV2, MobileNetV2 uses post-activation. Both down sampling and identity blocks consist of three sub-blocks. The first and last sub-blocks have convolution, batch-normalization, and ReLU activation layers. The second sub-block has depth-wise convolution, batch-normalization, and ReLU activation layers. The identity block has a skip connection from the input and is added to the output of the last ReLU layer. The first and last convolution has kernel size, *k* = 3, and stride, *s* = 1. The depth-wise convolution has stride, *s* = 2.

Lastly, we incorporate the EfficientNetV2 pre-trained encoder, which consists of Squeeze-and-excitation blocks. The block is illustrated in figure 4(v). The block consists of three sub-blocks, with the first and third consisting of convolution, batch-normalization, and Swish activation layers. The second sub-block consists of global-average pooling, convolution, Swish activation, convolution, and Softmax activation layers. The output of the second sub-block is elementwise multiplied by the first sub-block output. The convolution in the sub-block has a kernel size, *k* = 3, and the rest of the convolution in other sub-blocks has a kernel size, *k* = 1. As Efficient-NetV2 only utilizes this block for down-sampling and regular blocks, the stride size changes in the first sub-block depending on the block definition. The stride size is chosen as, *s* = 2 for down-sampling and *s* = 1 for regular block.

#### 2.4.3. Regression decoder

The decoder consists of a global average pooling layer and three dense layers, as illustrated in figure 3. The global average pooling takes the average of the spatial dimensions and transforms it into a 1D feature vector. The three dense layers utilize piecewise linear activation function [86] and consist of 256, 64, and 2 neurons. We use two neurons in the last layer for predicting *R*_0_ and *β*. The down-stream task is a supervised-regression task, as the ground-truth values of *R*_0_ and *β* are positive real numbers, *R* ⩾ 0 = {*xεR: x* ⩾ 0}.

#### 2.4.4. Objective function

To ensure that the *R*_0_ and *β* values are accurate in terms of prediction, we incorporate two mean-absolute error (MAE) losses for each of them, as shown in equations (13) and (14) [87]. Here, ${y_{{R_0}}}$ and ${y_\beta }$ are ground-truth values and ${y_{{{\hat R}_0}}}$ and ${y_{\hat \beta }}$ are predicted values. The *N* signifies the number of samples

\begin{equation*}{\mathcal{L}_{{R_0}}} = \frac{1}{N}\mathop \sum \limits_{i = 1}^N \left| {{y_{{R_0}}} - {y_{{{\hat R}_0}}}} \right|\end{equation*}

\begin{equation*}{\mathcal{L}_\beta } = \frac{1}{N}\mathop \sum \limits_{i = 1}^N \left| {{y_\beta } - \hat{y_\beta }} \right|.\end{equation*}

By incorporating equations (13) and (14), we can formulate our final loss function as given in equation (15) [88]. Here, *φ* is the weight for prioritizing individual loss functions.

We use *φ* = 0.5, as we want to prioritize both ${\mathcal{L}_{{R_0}}}$ and ${\mathcal{L}_\beta }$ losses


\begin{equation*}\mathcal{L} = \phi {\mathcal{L}_{{R_0}}} + \left( {1 - \phi } \right){\mathcal{L}_\beta }\,.\end{equation*}

Additionally, for convenience, all the mathematical parameters used in equations (1)–(15) have been summarized with symbols and explanations in the supporting section, table S-2.

## 3. Results and discussion

In the results section, we present a simplistic way to measure the surface tension of pendant droplets from a deep-learning model. We explain how we have trained our models from the LM data set and report the corresponding parameters during the training process. Our models could predict the drop parameters from the pendant drop image with high accuracy. Then, we present our models’ utility by calculating the surface tension value of perturbed droplets that were not calculable with the DropImage software [43]. Our models are made as versatile models capable of measuring the drop shape parameters directly from the pendant drop image. Measuring the shape parameters will allow our models to work with other fluid droplets. By incorporating this deep learning analysis, pendant drop techniques can be used to measure the dilatational modulus of LM in different mediums. At the end of this result section, we present the elastic and loss modulus investigation of LM droplets in the air.

### 3.1. Deep learning analysis

#### 3.1.1. Hyper-parameters

For training the Deep regression model, we used the Adam optimizer for the supervised regression head, with the initial learning rate set to *lr*= 0.0001. We used an adaptive learning scheme of updating the weights if the validation loss does not decrease for six successive epochs. The learning rate would be decreased according to the equation *lr*= *lr* ∗ *λ*, where *λ* = 0.1. We use a mini-batch size of 8 and train for 30 epochs.

#### 3.1.2. Quantitative evaluation

To effectively measure the model’s performance, we utilize three metrics, (i) MAE, (ii) Mean-squared-error (MSE), and (iii) *R*-squared score. As seen in table 1. Our best model, EfficientNet-v2, achieved a 99.97% *R*-squared score, 4.51 × 10^−4^ MAE, and 4.73 × 10^−7^ MSE for ${R_0}\,$ values. Compared to that, ResNet50-v2 achieved almost 0.49% less *R*-squared score and higher MAE and MSE. Similarly, MobileNet-v2 achieved a 0.03% worse *R*-squared score and higher MAE and MSE. Similarly, EfficientNet-v2 outperformed ResNet50-v2 and MobileNet-v2 for *β* and *γ* values across all three metrics, as seen in tables 2 and 3. Aside from the partial differences in *R*-squared accuracy, all three of our models achieved over 99% *R*-squared value, which means our model can explain variance for more than 99% of samples. One noticeable thing in the tables is that the MSE and MAE values for *β* and ${R_0}$ have minor differences, and *γ* has large error margins. However, the actual values of *R*_0_ are between 1.0 and 1.2, and *β* is between 0.08 and 0.22. On the other hand, the values for *γ* are between 300 to 800. The differences are stark as we did not normalize the MAE and MSE metrics as percentages and kept the raw values.

**Table 1.** Evaluation of *R*_0_ values on test images.

Encoder	Mean-absolute-error	Mean-squared-error	R-squared score
ResNet50-v2	3.15 × 10^−3^	1.03 × 10^−5^	99.48%
MobileNetV2	8.34 × 10^−4^	1.15 × 10^−6^	99.94%
EfficientNetV2	4.51 × 10^−4^	4.73 × 10^−7^	99.97%

**Table 2.** Evaluation of *β* values on test images.

Encoder	Mean-absolute-error	Mean-squared-error	*R*-squared score
ResNet50-v2	6.05 × 10^−4^	6.24 × 10^−7^	99.93%
MobileNetV2	5.61 × 10^−4^	7.86 × 10^−7^	99.92%
EfficientNetV2	4.15 × 10^−4^	5.28 × 10^−7^	99.94%

**Table 3.** Evaluation of *γ* values on test images.

Encoder	Mean-absolute-error	Mean-squared-error	R-squared Score
ResNet50-v2	1.442	6.001	99.67%
MobileNetV2	1.2172	4.833	99.91%
EfficientNetV2	0.9945	3.949	99.97%

**Figure 5.**
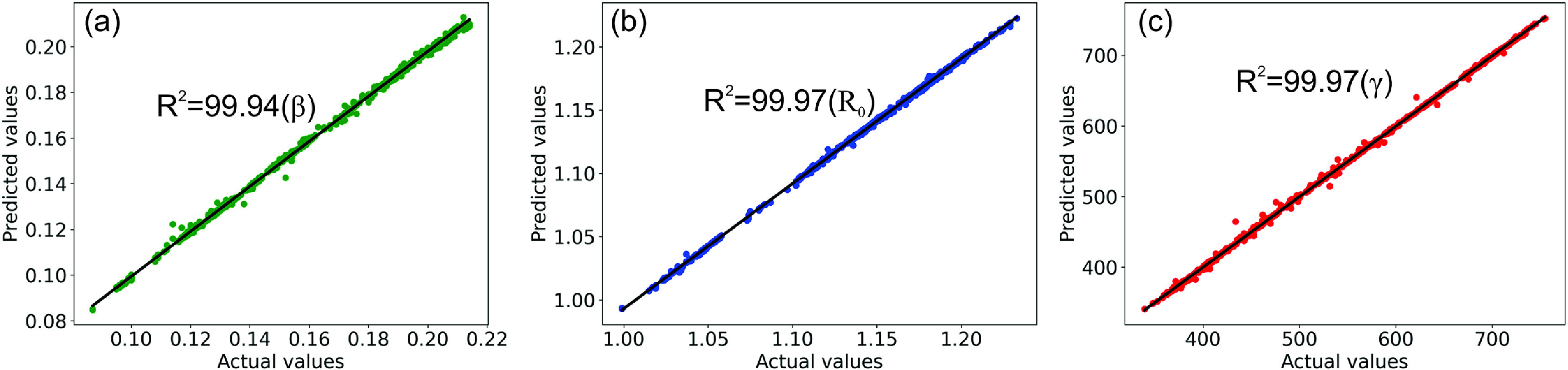
Predicted vs. actual values plotted for $\beta ,{R_0}\,$, and $\gamma $ in figures 1(a)–(c). For all the figures, we want the intersection (green dots for $\beta $, blue dots for$\,{R_0}$, and red dots for $\gamma $) of the predicted value (*y*-axis) and actual values (*x*-axis) to be diagonally placed (diagonal black line). The more the different dot deviates from the diagonal black line, the worse the results, whereas the closer it is to the black line, the better the predicted results. As illustrated in all three figures, our predicted values are quite accurate for $\beta ,{R_0},\,$ and $\gamma $ value prediction.

We also demonstrate its effectiveness, as illustrated in figure 5. Here, the *x*-axis is the ‘Actual values,’ and the *y*-axis is the ‘Predicted values.’ The different colored dots are predicted values, and the diagonal line in solid black represents the correct prediction. The deviations between the predicted and the actual values are measured through the statistical variance, ${R^2}$. Please note that the predicted values are obtained from our ML model, and the actual values are measured using the DropImage software. For shape parameter *β,* the deviation was 0.06%, and for radius, ${R_0},$ the deviation was 0.03%. The predicted surface tension value, *γ*, showed 99.97% accuracy and demonstrates effectiveness in measuring the axisymmetric surface tension value through our methodologies. As seen in the figures *R*_0_, *β*, and *γ*, the predicted values have minor deviations compared to actual values.

### 3.2. Prediction of highly distorted shape

**Figure 6.**
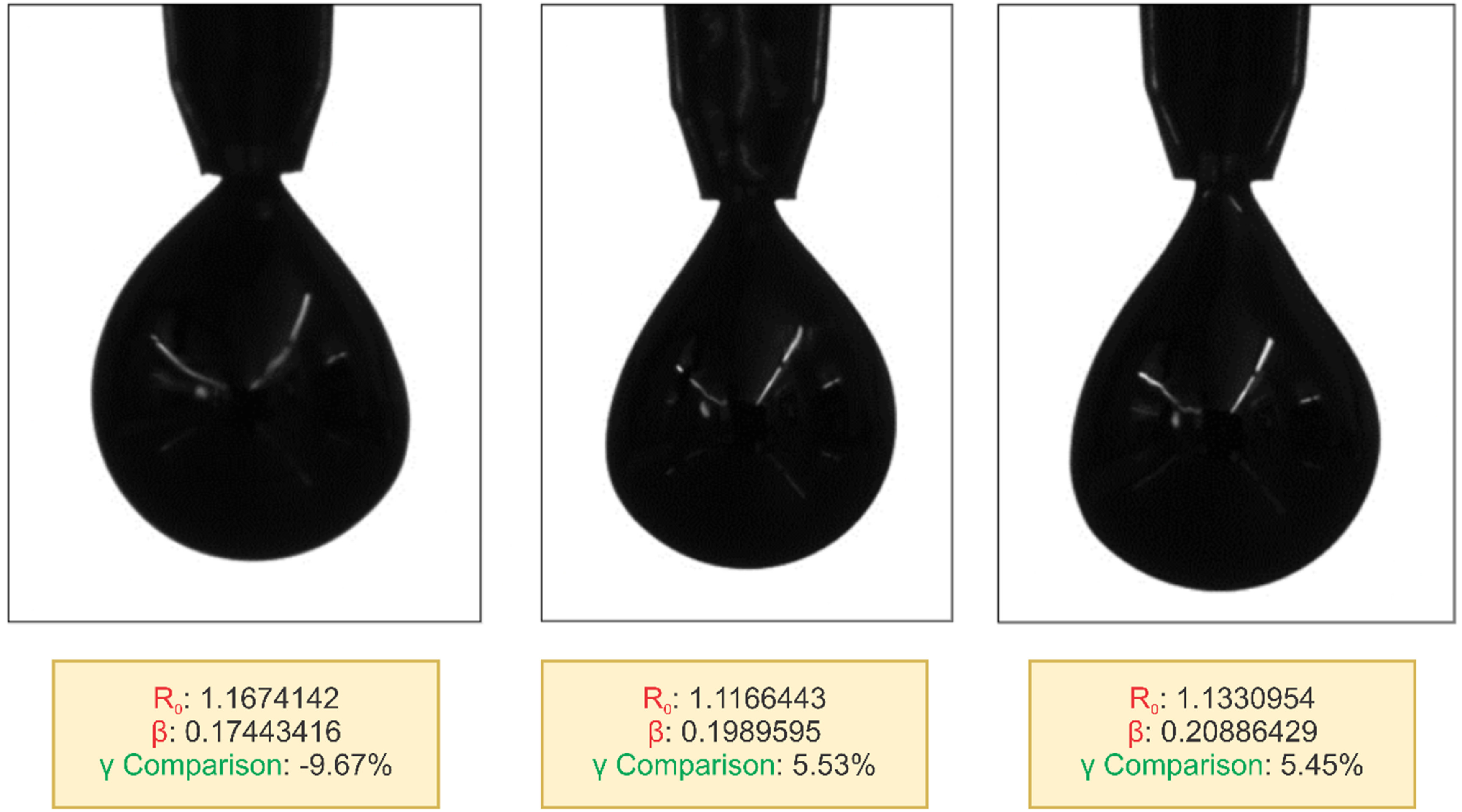
Prediction for ${R_0},\,\beta $ for perturbed pendant drop images using the deep learning approach in this work. Using these parameters in the young-Laplace equations gives an approximate interfacial surface tension, *γ*, compared with the values from a manual open-source software [44]. As the software uses the axisymmetric assumption, the detected edge in the manual measurement shown in the supporting figure S3, does not truly represent the actual perturbed shape.

We also tested our model’s effectiveness with distorted pendant drop images. In a standard setting, these images cannot be analyzed, and any *β, γ*, or ${R_0}$ values are not achievable from the traditional software [43]. Instead, it cannot detect proper symmetry along the central axis and throws an error due to its distorted or perturbed shapes. As shown in figure 6, our model can effectively produce approximate values for these images. Our models have been trained with continuous pendant shape values and can interpolate the values for distorted shapes. We have validated the results by manually calculating the approximate value using open-source software [44]. For all these scenarios, our model successfully predicted the approximate value. We also need to remember that the surface tension value calculated by the OpenDrop software [44] is not exactly accurate as it tries to fit an axisymmetric shape to the perturbed shape [see figure S-3]. This software requires a manual declaration of the tip length and drop shape, which is significantly time-consuming. However, once trained, our model can quickly predict these values. Combining pendant-sessile methods or membrane or shell impacts can help determine these droplets’ proper surface tension value [89–91]. We want to extend our work in the future to validate these results. Additionally, as the axisymmetric assumption does not hold for fluids like LM, it needs to be remembered that the pendant droplets may show different states from another perspective. For example, while an LM pendant droplet shows symmetry from the front (camera perspective of the goniometer), it may have a distorted shape on other sides (side view or the opposite of the camera perspective) or vice versa. Investigating from a 3D perspective was beyond the scope of this work, and we aim to contribute to this in our future work.

### 3.3. Tensiometry analysis

**Figure 7.**
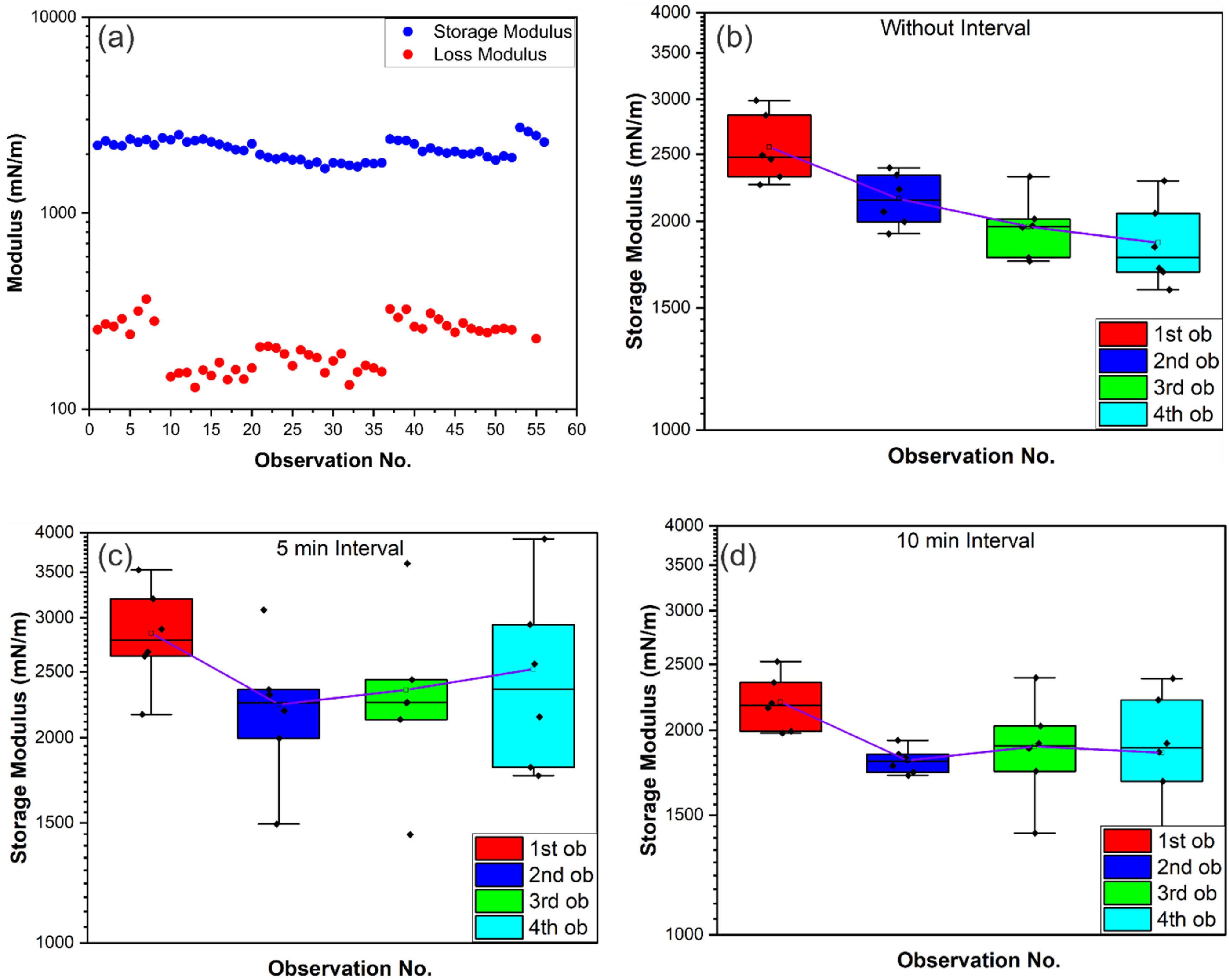
(a) comparison of storage (elastic) and loss (viscous) modulus of LM in air medium, the behavior of LM in air medium showing (b) significant reduction of storage modulus for continuous observation, (c) initial reduction and slight recover of the storage modulus for 5 min interval between observations, (d) reduction and plateauing of the storage modulus for 10 min interval between observations.

In conjunction with our findings on surface tension, we also investigated oscillating pendant droplets to evaluate dynamic moduli. We measured the storage and loss modulus of LM in native air to establish the basis for utilizing pendant droplets for dynamic studies. The experimental setup is shown in figure 1, and the procedure has been explained in the materials and methods section. For all cases, we found that the storage modulus was at least one-fold higher than the loss or viscous modulus shown in figure 7(a). This phenomenon is also expected as the viscous component comes from the nano-layer of gallium oxide [14], which is insignificant to the total volume of the pendant droplet. The storage modulus is expected to come from the bulk LM. All the previous works have also reported that storage modulus dominates the viscous modulus of LM similarly in air. Researchers have investigated the modulus using different rheometers based on different working principles but still have reported a similar difference between the modulus [12, 14, 27, 62]. As the volume is constantly oscillating in a short range [cf figures 7(b)–(d)], the oxide layer is continuously evolving (either rupturing or stretching into new oxides) [24, 92] as the yielded surface. It is noted that a low ∼0.5 N m^−1^ [12] surface stress is required to yield the oxide, exposing the bulk metal to a ‘different shades of oxide’ [93] so that a new interfacial interaction can be induced.

This pendant droplet tensiometry provides the opportunity to investigate this continuous interaction with air. We oscillated the same droplet with different intervals between the oscillation. We oscillated each droplet four times and repeated the experiments with several droplets of LM with similar sizes. When the interval between two oscillations was not present (there was a slight unmeasurable interval due to the instrumental data capturing and processing limitation), we saw a decrease of the modulus in figure 7(b), which indicates the oxide becomes ‘softer.’ More LM on the surface portion may convert into the viscous Ga_2_O_3_, reducing the modulus [14]. When 5 min interval was introduced between observations, a significant drop can be seen between the 2nd and 1st observation, similar to the difference between the 4th and 1st observation in figure 7(a). Then, the modulus starts to increase slightly over time for 5 min interval situations. The softening effects almost plateaued over time when the interval increased to 10 min in figure 7(d). However, the large data distribution for the 3rd and the 4th observations in 5 and 10 minute intervals indicates more random behavior for long interaction of LM with Air. To summarize, a decrease in modulus also suggests that the state of the surface oxide between oscillations changes, and the immediate or intermediate surface states can be assessed, harnessing the oscillation-induced tensiometry analyses.

## 4. Conclusion

Surface tension holds an essential role in the application of fluid. Conventional theories and methods for measuring the surface tension of fluids are based on the assumption of axisymmetrical pendant droplets. However, LM’s unique reactive oxide layer can lead to non-axisymmetrical pendant droplets. Commercial software often produces error values and ignores the surface tension values of these perturbed droplets. This obstacle has not allowed the Pendant drop-based oscillatory tensiometry technique, a convenient and facile technique for dilatational modulus investigation, to investigate the LM oxide layer. To overcome this challenge, we proposed a deep-learning method for predicting the intermediate surface tension of these distorted droplet shapes. Our models are also universal enough to work with pendant droplets of other fluids. Unlike previous ML models that use classification techniques, our regression-based model can predict the continuous value of surface tension. Our study created a dataset of LM droplets with varying surface tension to train our deep learning. Our models have shown high accuracy in predicting the pendant droplet’s drop-shape parameters. It has the versatility to work for other fluids also. We have also showcased our ML approach’s direct utility in predicting the approximate surface tension value of the nonaxisymmetric pendant droplets of LM. We further harnessed the technique to measure the elastic and viscous modulus of LM in the air medium with an oscillatory pendant drop technique. We have found dominance of elastic modulus over viscous modulus. We also reported the softening behavior of LM droplets due to dynamic interaction with air. This bubble tensiometry or oscillatory pendant drop technique will equally apply to investigate the LM oxide interaction with water, acid, base, and other polymers- however, for the current study, we have kept our demonstration only in native air. Future investigations can be done in other media. We believe our current study will create a new horizon in materials science where researchers from ML and artificial intelligence domains can work synergistically to solve more complex problems related to surface science, interfacial studies, and other studies relevant to LM-based systems.

## Data Availability

All data that support the findings of this study are included within the article (and any supplementary files). The image dataset for training the ML model are available from the corresponsing author upon reasonable request.
